# Patients’ priorities in kidney stone disease

**DOI:** 10.1308/rcsann.2025.0051

**Published:** 2025-07-15

**Authors:** V Popoola, G Wheeler, SA Howles, CE Lovegrove

**Affiliations:** ^1^ University of Oxford, UK; ^2^ Oxford University Hospitals NHS Foundation Trust, UK

**Keywords:** Urolithiasis, Patient and public involvement, Therapeutics

## Abstract

**Introduction:**

Engaging with patients and the public is essential to design and deliver impactful research. Enhancing the relevance of research and tailoring treatments to align with patients’ preferences can facilitate improved clinical care.

**Methods:**

We aimed to identify the research, support and treatment priorities of individuals with kidney stone disease (KSD) using a 25-question survey in inpatient and outpatient urology departments.

**Results:**

Forty-four individuals with KSD responded to our survey; 28 (64%) had experienced multiple KSD episodes and 11 reported 5 or more episodes. Median self-rated quality-of-life (QoL) impact (0 = negligible; 10 = severe) was 7.00 out of 10.00 (interquartile range [IQR]: 5.00–9.00), equivalent in individuals with single and recurrent stone episodes. Pain (*n* = 34), haematuria (*n* = 28) and anxiety (*n* = 22) were the primary factors contributing to QoL impact. Participants prioritised research into preventing recurrence, alleviating pain and slowing stone growth. More than one-third desired more information about KSD. Most (*n* = 36) felt ‘likely’ or ‘very likely’ to take medication to reduce their risk of KSD and 25 would commit to life-long therapy. Daily dosing was acceptable to 13 participants if risk of KSD recurrence was reduced by 50%, rising to 34 respondents if risk of recurrence was reduced by 75%. Most respondents (*n* = 44) expressed willingness to have genetic testing to facilitate personalised medicine research.

**Conclusions:**

Our findings emphasise symptoms contributing to reduced physical and psychological wellbeing in patients with KSD. We highlight the need for research into developing therapies to prevent stone recurrence, alleviate pain and slow stone growth, and for educational materials. Responses indicate an appetite for personalised medicine and oral medications in KSD.

## Introduction

Kidney stone disease (KSD) affects ∼15% of the world's population and is a significant cause of morbidity, accounting for >40,000 interventions, >88,000 acute hospital admissions and ∼130 deaths each year in the United Kingdom (UK).^1^ KSD has a profound impact on quality of life (QoL) because kidney stones may cause recurrent episodes of acute pain, chronic pain, obstructive uropathy, urinary tract infections, haematuria and the need for surgical intervention.^3,4^ It is increasingly recognised that this impact is not solely physical; anxiety and depression are among the diverse psychological challenges reported by patients in the literature.^5^ Moreover, KSD carries a significant economic burden at individual and societal levels. Patients must manage the financial repercussions of transport and prescription costs in addition to absence from paid employment. In the UK, for the National Health Service (NHS), each acute stone-related episode costs £1,277 to £2,887 and England will spend more than ∼£300 million per year on KSD by 2030, which is comparable with the UK-wide cost of initial bladder and prostate cancer treatment combined.^6^

Despite the considerable physical, psychological and financial burden of KSD, the prevalence continues to increase and up to 50% of individuals who have had a stone will experience a second stone within 10 years.^7^ This recurrence is likely attributable to the continued lack of effective treatment options to reduce the risk of stone formation.^8–10^ At present, lifestyle advice forms the mainstay of KSD management and encompasses increasing water intake, reducing salt intake, avoiding carbonated drinks, adding fresh lemon juice to drinking water, and maintaining a normal daily calcium intake. Given the well-documented challenge with compliance and the significant changes we ask patients to make in their day-to-day lives, extensive efforts have gone into identifying alternative therapeutic strategies including prophylactic medications to reduce KSD recurrence.^9,11–13^ However, at present, there is a paucity of data describing patients’ concerns and unmet needs and their priorities for research and what treatments they might find acceptable.^14–16^ Given that patient and public involvement is crucial to augmenting the quality, relevance and clinical impact of research, we sought to: (i) identify the research, support and treatment priorities of individuals with lived experience of KSD; (ii) ascertain whether patients would be interested in a prophylactic medication that aims to reduce KSD recurrence; and (iii) identify factors, such as dosing regimen and side effect profiles, that might influence this interest.^17,18^

## Methods

### Ethics approval

This project was registered with Oxford University Hospitals (project number 8688). The survey and methods were reviewed by the Joint Research Office study classification group, a collaboration with the Oxford Health NHS Foundation Trust and the University of Oxford, and were judged to not require sponsorship or further research ethics review (Appendix 1 ­– available online).

### Participants­

Participants were invited on a voluntary basis from adult (>18 years of age) inpatient and outpatient urology departments in a single tertiary centre in the UK between April 2023 and January 2024. Other than limiting responses to only include patients who had experienced a kidney stone, there were no further inclusion or exclusion criteria.

### Questionnaire overview

The primary aim was to elucidate the research, support and treatment priorities of patients with KSD. A 25-question survey, comprising closed and open-ended questions was designed to identify the treatment priorities of patients affected by KSD (Appendix 2 – available online). Our primary aim was to elucidate the research, support and treatment priorities of patients with KSD rather than assess QoL. In light of this, we did not use the entirety of the validated Wisconsin Stone Quality of Life questionnaire to avoid participants experiencing survey fatigue, but incorporated a subset of the QoL questions into our survey.^19^ The survey covered three main domains: patient experience of KSD; willingness to use prophylactic medication; and priorities in treatment, support and research. Because our research group aims to progress understanding of the genetic pathophysiology of KSD and to identify new therapeutic opportunities to prevent kidney stone recurrence, we also explored patients’ willingness to undergo genetic testing to facilitate future personalised therapeutic trials. The questionnaire included multiple choice and Likert scale questions, with opportunities for free-text responses and was accessed via a quick response (QR) code from posters in inpatient and outpatient urological departments in a single urology unit between April 2023 and January 2024 (Appendix 3 – available online).

Respondents were categorised as individuals who had experienced a single kidney stone episode or those who had had recurrent KSD (two or more episodes at least 6 months apart). The impact of KSD on patients’ QoL was scored on a Likert scale from 0 to 10, inclusive (0 = negligible impact, 10 = the most severe impact possible) (Appendix 1 – available online). For two questions (Which symptoms have you experienced? and Which treatments have you undergone?), participants could select as many answers as were applicable, thus the number of responses exceeds the sample size. Willingness to take prophylactic medication was assessed using a Likert scale ranging from ‘very likely’ to ‘very unlikely’ (Appendix 1 – available online). Perceived importance of alleviating pain, slowing stone growth and reducing stone recurrence were appraised using a Likert scale from 0 to 10 (0 = not at all important, 10 = very important).

After publicising the questionnaire, we noticed that the wording for multiple choice options in question 16 (Which of the following do you think could be helpful to support people with kidney stone disease?) was unclear. Thus, we do not report responses to this question.

### Statistical analysis

Descriptive statistics (mean, standard deviation [sd], median and interquartile ranges [IQR]) were generated to represent the characteristics of the surveyed population. Statistical analyses were undertaken to test the null hypothesis that there was no significant difference in responses between individuals who had experienced a single stone episode and those who had experienced recurrence. Wilcoxon rank sum test with continuity correction was used for continuous variables that were not normally distributed and Pearson's chi-squared test was used to compare proportions. All statistical tests were two-sided, and a *p-*value of <0.05 was considered significant after adjusting for multiple testing using the Benjamini–Hochberg false discovery rate method, controlled at 5%.^20^ Statistical analyses were performed using the dplyr package for R software and graphs were generated in Excel.^21^ Free-text responses were thematically categorised by the authors manually.

## Results

### Patient population

**Figure 1:**
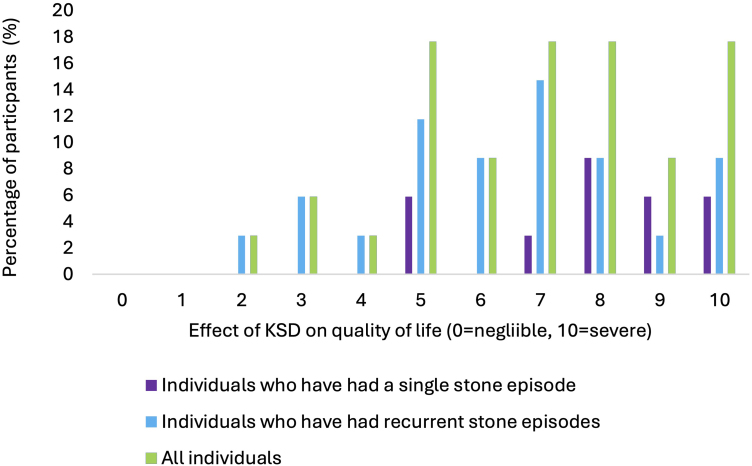
The impact of kidney stone disease on self-reported quality of life in all individuals (green), individuals who have had a single stone episode (purple) and individuals who have experienced recurrence (blue) Wilcoxon rank sum test with continuity correction was used for statistical analysis; *p* < 0.05 is considered significant. There was no significant difference between single and recurrent stone formers, *p* = 0.61.

Over 9 months, 44 individuals with KSD responded to the survey. Of these, 28 had experienced multiple KSD episodes and 11 reported 5 or more episodes (Table 1). The most common treatment strategy that respondents had undergone was ‘watch and wait +/− tamsulosin’ (*n* = 23) (Table 1). Of the 34 respondents who answered the QoL question, the median score (0 = negligible, 10 = severe) for the effect of KSD on an individual's QoL was 7.00 out of 10.00 (IQR 5.00–8.00). This was equivalent for individuals with single and recurrent stone episodes (8 of 10 [IQR 7.00–9.00] in single stone formers; 7 of 10 [IQR 5.00–8.00] in recurrent stone formers; *p* = 0.61) (Figure 1).

**Table 1:** Baseline characteristics of survey participants

	No. of respondents (%)
No. of previous stone episodes	
1	14 (32)
2	10 (23)
3	3 (6.8)
4	4 (9.1)
>4	11 (25)
No response	2 (4.5)
Past interventions received	
Watch and wait (with or without tamsulosin)	23 (52)
Lithotripsy	16 (36)
Stent insertion	10 (23)
Ureteroscopy and laser destruction of stone	13 (30)
Percutaneous nephrolithotomy	6 (14)
Percutaneous nephrostomy	2 (5)
No response	10 (23)
KSD symptoms experienced	
Side effects from antibiotics given for KSD	9 (20)
Infection	15 (34)
Low mood	14 (32)
Anxiety or fear about future kidney stones	22 (50)
Haematuria	28 (64)
Stent-related symptoms	1 (2)
Pain from kidney stone treatment	19 (43)
Pain from a kidney stone	34 (77)
Most bothersome symptom from stone/stone-related treatment	
Pain from a kidney stone	28 (64)
Pain from kidney stone treatment	0 (0)
Stent symptoms	0 (0)
Haematuria	0 (0)
Anxiety about future kidney stones	4 (9.1)
Low mood	0 (0)
Infections	2 (4.5)
No response	10 (23)

KSD = kidney stone disease

Respondents (*n* = 44) were asked to report the number of previous stone episodes experienced, the past interventions they had received, the symptoms they had experienced and the most bothersome symptom that they had experienced. Respondents were permitted to select as many treatments and symptoms as were applicable, thus reported percentages do not add up to 100%.

Pain from a kidney stone (*n* = 34), haematuria (*n* = 28) and anxiety about recurrence (*n* = 22) were the most reported factors contributing to QoL impact (Table 1). Of these symptoms, pain was the most bothersome across the cohort (*n* = 28) (Table 1).

### Willingness to take prophylactic medication

**Figure 2:**
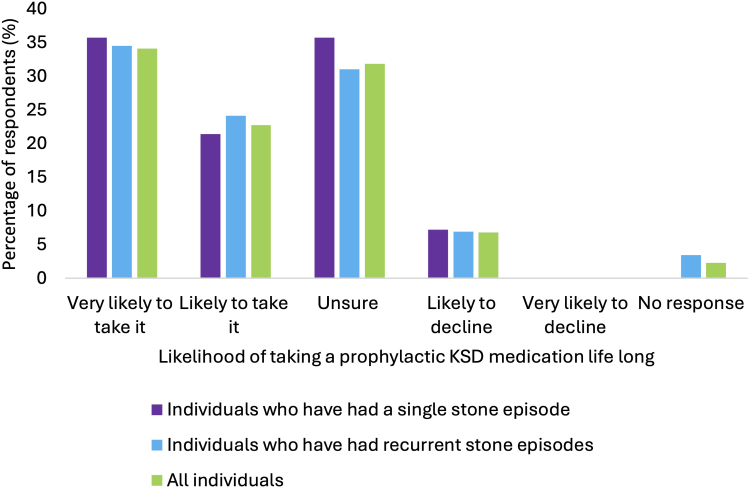
Likelihood of taking a life-long medication that would reduce the chance of stone formation in all individuals (green), individuals who have had a single stone episode (purple) and individuals who had experience recurrence (blue) Respondents were given a range of options to describe their likelihood of taking life-long prophylactic medication for kidney stone disease. Pearson's chi-squared test was used for statistical analysis, *p* < 0.05 is considered significant. There was no significant difference between single and recurrent stone formers (*p* = 0.97).

**Figure 3:**
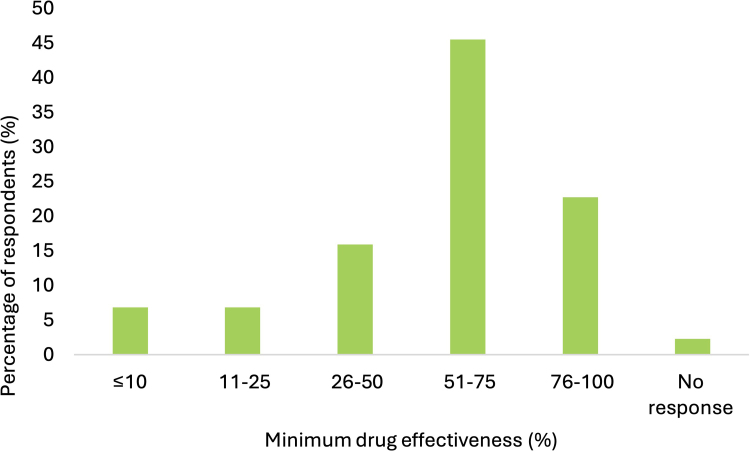
Minimum effectiveness of a drug in preventing risk of recurrence to merit daily dosing

Most (*n* = 36) respondents felt ‘likely’ or ‘very likely’ to take a medication to reduce their risk of stone formation (12 of 14 in single stone formers and 24 of 30 in recurrent stone formers; *p* = 0.98) (Figure 2); 57% (*n* = 25) felt willing to commit to life-long therapy (8 of 14, in single stone formers and 17 of 29 in recurrent stone formers; *p* = 0.97) (Figure 2). Only 6.8% (*n* = 3) were likely or very likely to decline medical therapy. A once-daily dosing regimen was acceptable to 86% (*n* = 38) of respondents, with a more frequent regimen acceptable to 45% (*n* = 20) (Table 2). Daily dosing was acceptable to 30% (*n* = 13) of participants if risk of KSD recurrence was reduced by 50%, rising to 77% (*n* = 34) respondents if risk of recurrence was reduced by 75% (Figure 3).

**Table 2:** Maximum number of times a day respondents were willing to take a prophylactic medication for kidney stone disease

Maximum number of times a day respondents are willing to take a medication which reduce kidney stone disease	No. of respondents (%)
Less than once daily	6 (14)
Once daily	17 (39)
Twice daily	11 (25)
Three times daily	5 (11)
Four times daily	4 (9.0)
No response	1 (2.0)

After considering the possibility of mild side effects (nausea, diarrhoea, change in urination frequency), 30% (*n* = 13) of respondents felt ‘likely’ or ‘very likely’ to use prophylactic medication (4 of 14 in single stone formers and 9 of 30 in recurrent stone formers; *p* = 0.97). In free-text boxes, nausea, diarrhoea, increased urinary frequency and effects on fertility or potency were described as side effects that would prevent patients from taking prophylactic medication.

### Patient priorities for treatment, support and research

Respondents felt strongly that treatment for individuals with recurrent KSD should be improved, rating its mean importance as 9.1 of 10 (sd = 1.2). Participants indicated that efforts to improve the therapies available to patients with KSD should focus on alleviating pain, reducing recurrence rates and reducing stone growth, with mean importance ratings of 9.8 of 10 (sd = 0.81), 9.7 of 10 (sd = 1.1) and 9.5 of 10 (sd = 1.8), respectively. When asked what provisions could be helpful to people with KSD, 75% (*n* = 33) selected answers that involved greater support, ranging from telephone advice to support groups, and more than one-third (*n* = 16) desired more readily accessible information about KSD for patients, perhaps in the form of booklets or websites.

The free-text responses to the question ‘Is there anything you would like to see prioritised in kidney stone research?’ fell into eight broad categories, in order of frequency: (i) understanding the role of diet in causing KSD; (ii) preventing recurrence of KSD; (iii) improving pain management; (iv) furthering the understanding of the aetiology of KSD; (v) developing effective pharmacological therapies; (vi) understanding the influence of KSD on other diseases; (vii) improving the detection of KSD; and (viii) surgical interventions for KSD. The responses highlighted that, while research should prioritise understanding the aetiology of disease and how it can be prevented, patients also desire more streamlined handling of acute stone episodes from diagnosis to management. For example, many responses detailed the difficulties patients faced when attending primary and secondary care settings with symptoms of renal colic, describing waiting in agony for many hours, only to receive ineffective analgesia or to undergo inadequate imaging modalities. This is a stark reminder of the significant impact that KSD has on individuals. Almost all respondents (93%, *n* = 41) expressed willingness to have their DNA tested should there be a trial to elucidate whether personalised medicine is a viable therapeutic approach in KSD; results were comparable between single and recurrent stone formers (13 of 14 vs 28 of 30, respectively; *p* = 0.97).

## Discussion

To our knowledge, this is the first analysis exploring the research, support and treatment priorities of patients with KSD in the UK. Overall, patients want improved access to information about their condition, improved support and management during acute stone episodes, and are willing to take prophylactic medication to reduce kidney stone recurrence. Results from this survey should be used to develop strategies to enhance the clinical pathways for patients with KSD and to inform research efforts aimed at improving the therapies available to them. A recent study, conducted in the United States, has prioritised research topics in KSD by engaging stakeholders including patients, caregivers, clinicians and researchers.^22^ The findings, which highlighted key themes of genetic evaluation, cumulative disease burden and psychosocial support, align with some of the priorities identified in our survey. However, our analysis offers a more focused exploration of the primary concerns directly from the patients’ perspective. In addition, we provide valuable insights into what patients consider acceptable therapeutic options for KSD, harnessing the unique viewpoint that they offer. By leveraging these findings, it may be possible to reduce the burden of KSD on both patients and healthcare providers.

### Patients’ research, support and treatment priorities

Disease prevention is a key theme that emerged from free-text responses to the question ‘Is there anything that you would like to see prioritised in kidney stone research?’. Currently, there is no high-quality evidence to support dietary or medical interventions to prevent kidney stone recurrence.^8–10^ Our data indicate that most patients feel positively about prophylactic medications and more than 80% of individuals would be ‘likely’ or ‘very likely’ to take a drug that would reduce the risk of KSD.

When asked ‘How you think we should support patients and improve treatments for kidney stone disease?’, more than one-third of participants expressed a desire to receive more readily accessible information about their condition. In free-text responses, respondents expressed their uncertainty on what causes kidney stones and whether dietary prevention strategies would be beneficial, implying that patients are willing to engage in lifestyle approaches to prevent KSD and to learn about the aetiology of their disease. This may reflect inadequacies in the health education provided by encounters with healthcare staff. In the age of digital media, a range of patient information leaflets are available from the American Urology Association, European Association of Urology and the British Association of Urological Surgeons – a widely used resource for patients and urology practitioners in the UK – explaining what KSD is and lifestyle modification and treatment options for KSD.^23–26^ Thus, there is scope to review and improve the availability and application of existing educational resources for patients. Although information from leaflets would beneficial, alternative forms of media, such as educational videos about KSD, may bring greater patient satisfaction, as described by Fenn and McGuire.^27^ With the changing digital landscape and stones increasingly affecting younger people,^7^ the urological community should aim diversify the platforms through which patient information is available.

### Patients’ willingness to take prophylactic medication

Patient adherence with therapeutic recommendations from urologists or primary care providers is an appreciable challenge when managing KSD.^11^ Thus, it is essential to establish patients’ views on what constitutes an acceptable dosing regimen and side effect profile when considering developing or prescribing future pharmacological therapies. Our analyses, perhaps unsurprisingly, describe that the more a medication could reduce the risk of KSD, the more patients would accept daily dosing. Recent data – most prominently the NOSTONE trial – have put the effectiveness of thiazide diuretics in treating KSD into question, hence potassium citrate may be considered the most promising prophylactic medication currently available for KSD.^28^ However, our analyses suggest that the dosing regimen and side effect profile of potassium citrate is not deemed acceptable by patients, in keeping with previously reported data.^11^ Our results suggest that a once-a-day dosing regimen would be acceptable to ∼80% of individuals with kidney stones, but fewer than 50% would accept more frequent dosing. Future and existing research strategies must prioritise developing novel therapies that align with patients’ preferences for high efficacy and minimal side effects.

When asked about personalised medicine, almost all respondents expressed willingness to have DNA testing to facilitate research into treatments for KSD. Our understanding of the genetic basis of KSD has progressed in recent years and personalised therapies that target specific biological pathways may soon become applicable.^29–31^ Our results demonstrate the willingness of patients to explore these approaches.

### Impact of KSD on quality of life

Our findings support the existing literature, showing that KSD exerts a profound impact on patients’ QoL, with a median reported score of 7.00 out of 10.00. However, only a subset of themes relevant to the Wisconsin Stone Quality of Life survey was included in our questionnaire, which may limit the generalisability of our results because our main aim was to elucidate the research, support and treatment priorities of patients with KSD. Health-related QoL is poor in individuals with kidney stones in comparison with the general population. This trend is attributable to both the acute symptoms experienced during a stone episode, such as pain and haematuria, and the longer-lasting effects of anxiety and low mood that are increasingly acknowledged as problems affecting patients with KSD.^5,32,33^ A meta-analysis by Neill *et al* demonstrated an association between anxiety and urolithiasis, with greater than expected numbers of KSD patients experiencing psychological distress.^34^

Previous studies have found that patients who have experienced more stone episodes and more KSD-related procedures report lower QoL scores than those who have experienced fewer stones.^33^ Interestingly, the results from our survey do not reflect these findings. Although our small sample size may be a contributing factor, we infer that KSD-related QoL is affected by symptoms other than those associated with an acute stone episode; for example, the longer-term effects of anxiety and low mood. These may arise from a single stone episode and can persist for months or years beyond the event. With recurrence rates of KSD rising it is unsurprising that these acute and chronic concerns reported by patients have not diminished for almost two decades, reflecting the failure of our specialty to instigate effective preventive measures.^35^ Thus, to address concerns expressed by patients with KSD we must explore preventive strategies in KSD.

### Study limitations

This single-centre survey had a small sample size, was only available in English, and only accessible via a QR code; thus, the results may not be generalisable to KSD patients from other geographical locations, cultural backgrounds or technical abilities. There remains an opportunity to elicit the KSD research, support and treatment priorities across other patient populations. In addition, given that respondents likely completed the questionnaire when in the urology department, answers may be biased to include patients who are more troubled by their symptoms and disease, limiting generalisability. However, findings from this survey corroborate findings from the literature, hence we believe that they provide a reliable framework with which to direct future research into KSD. Finally, the questionnaire does not distinguish between the impact of KSD-related symptoms and treatments during acute stone events vs interim periods, potentially influencing responses based on a patient's symptoms at the time of their response. Nevertheless, our identification of long-term effects on QoL, such as psychological impacts, highlights that the burden of KSD extends beyond acute episodes. Further research is needed to explore these temporal relationships with greater granularity.

## Conclusions

Involving patients and the public in research is vital to ensure that future studies are of high quality, are relevant, and will have clinical impact. This project has identified that patients’ priorities cover three broad areas: (i) improved access to information about KSD; (ii) better management and support during acute stone episodes; and (iii) a focus on developing better stone prevention strategies. Our work highlights unmet needs in holistic kidney stone care and provides insights into patient priorities for KSD research.

## Funding

S.A.H. is a Wellcome Trust Clinical Career Development Fellow and receives support from Kidney Research UK (RP_030_20180306), Oxford Biomedical Research Centre (NF-SI-0514–10091), and the Wellcome Trust (204826/z/16/z). C.E.L. is an M.R.C. Clinical Research Training Fellow (MR/W03168X/1).

## Rights retention statement

For the purpose of Open Access, the author has applied a CC BY public copyright licence to any Author Accepted Manuscript (AAM) version arising from this submission.
